# Identification of novel prognostic circRNA biomarkers in circRNA-miRNA-mRNA regulatory network in gastric cancer and immune infiltration analysis

**DOI:** 10.1186/s12864-023-09421-2

**Published:** 2023-06-13

**Authors:** Jianing Yan, Guoliang Ye, Yanping Jin, Min Miao, Qier Li, Hanxuan Zhou

**Affiliations:** 1 Department of Gastroenterology, The First Affiliated Hospital of Ningbo University, Ningbo, 315020 China; 2 Department of Pharmacy, Yinzhou Integrated TCM and Western Medicine Hospital, Ningbo, 315000 China

**Keywords:** Competing endogenous RNA, Circular RNA, Gastric cancer, Bioinformatics, Prognosis, Diagnosis

## Abstract

**Background:**

Gastric cancer (GC) carries significant morbidity and mortality globally. An increasing number of studies have confirmed that circular RNA (circRNA) is tightly associated with the carcinogenesis and development of GC, especially acting as a competing endogenous RNA for miRNAs.

**Objective:**

Our study aimed to construct the circRNA-miRNA-mRNA regulatory network and analyze the function and prognostic significance of the network using bioinformatics tools.

**Methods:**

We first downloaded the GC expression profile from the Gene Expression Omnibus database and identified differentially expressed genes and differentially expressed circRNAs. Then, we predicted the miRNA-mRNA interaction pairs and constructed the circRNA-miRNA-mRNA regulatory network. Next, we established a protein-protein interaction network and analyzed the function of these networks. Finally, we primarily validated our results by comparison with The Cancer Genome Atlas cohort and by performing qRT-PCR.

**Results:**

We screened the top 15 hub genes and 3 core modules. Functional analysis showed that in the upregulated circRNA network, 15 hub genes were correlated with extracellular matrix organization and interaction. The function of downregulated circRNAs converged on physiological functions, such as protein processing, energy metabolism and gastric acid secretion. We ascertained 3 prognostic and immune infiltration-related genes, COL12A1, COL5A2, and THBS1, and built a nomogram for clinical application. We validated the expression level and diagnostic performance of key prognostic differentially expressed genes.

**Conclusions:**

In conclusion, we constructed two circRNA-miRNA-mRNA regulatory networks and identified 3 prognostic and screening biomarkers, COL12A1, COL5A2, and THBS1. The ceRNA network and these genes could play important roles in GC development, diagnosis and prognosis.

**Supplementary Information:**

The online version contains supplementary material available at 10.1186/s12864-023-09421-2.

## Introduction

Gastric cancer (GC) was the fifth leading cause of cancer-related morbidity and the fourth leading cause of cancer-related mortality worldwide in 2021 [1]. Although increasing research has focused on treatment strategies, such as combination chemotherapy, abrogation of cholinergic input by vagotomy, and chemical denervation, the 5-year survival rate for advanced gastric cancer patients is still less than 5% [2, 3]. However, detection of GC at an early stage obviously increases the 5-year disease-specific survival rate to approximately 97–99% [4]. Current traditional tumor biomarkers, such as carcinoembryonic antigen (CEA), carbohydrate antigen 72 − 4, and gastrin-17, display a low positivity rate in GC screening [5, 6]. Meanwhile, as GC is a heterogeneous cancer, the treatment responses are difficult to predict and monitor [7]. Hence, it is critical to explore novel and satisfying methods to screen and monitor GC.

Circular RNAs (circRNAs) are a large class of endogenous RNAs with a closed circular structure generated by reverse splicing [8]. To date, the most well-established roles of circRNAs are as competing endogenous RNAs (ceRNAs) and as sponges for miRNA, and circRNAs are believed to be novel tumor regulators in tumorigenesis and carcinogenesis and are considered to be more effective than linear RNAs [9]. With the characteristics of abundance and stability, an increasing number of circRNAs have been identified as potential targets for disease diagnosis and treatment, providing a reference point for the study of malignant tumors. However, the functions of only a minority of circRNAs have been determined.

With the rapid technological breakthroughs of genome-wide microarrays and data mining, bioinformatics is providing insights for cancer diagnosis, grading and prognosis prediction. More importantly, bioinformatics can effectively address the problem of special disease and insufficient sample sizes in reality, which is helpful to determine tumor targets and elucidate the pathogenesis of malignant tumors [10].

**Fig. 1:**
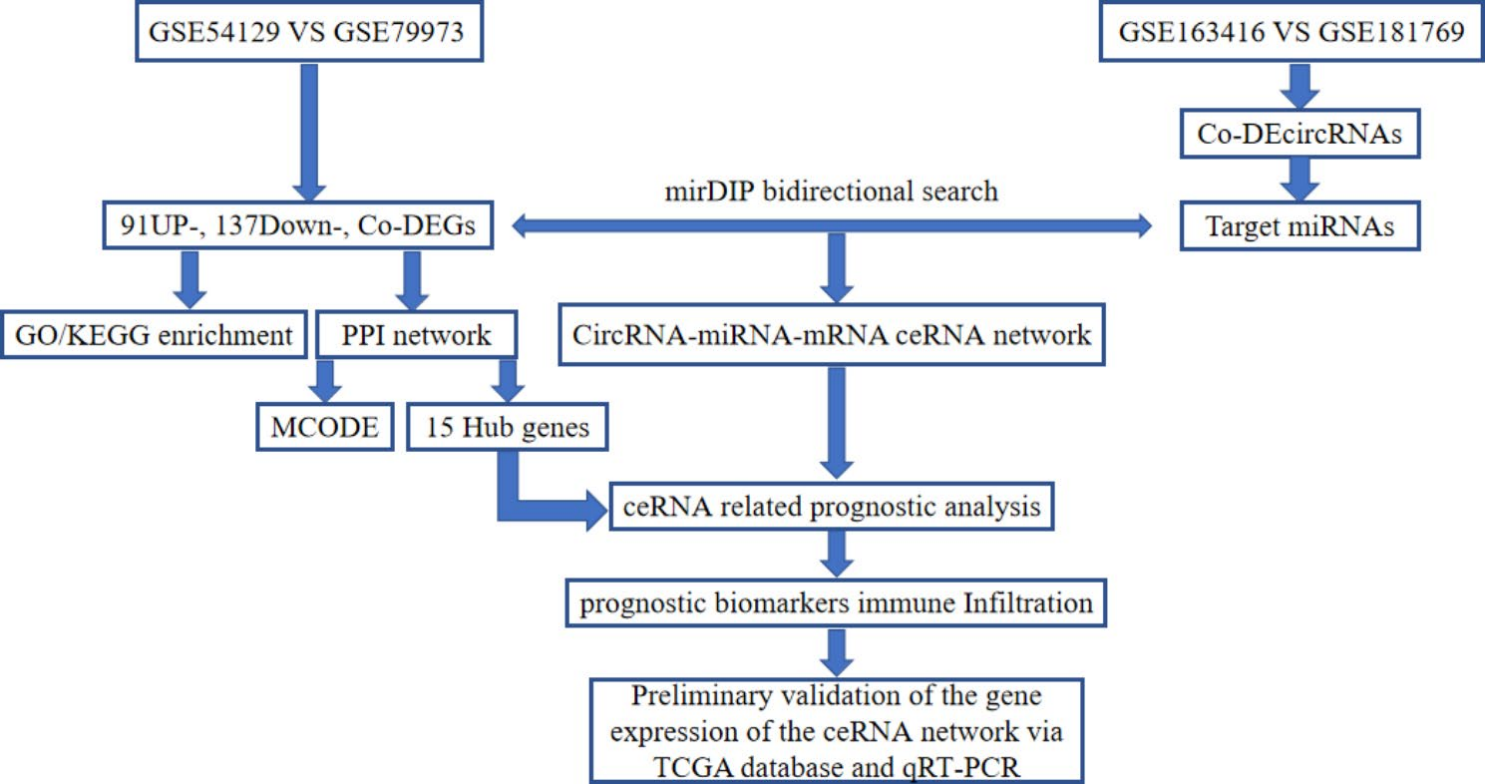
Flow chart of comprehensive bioinformatics analysis in establishing the circRNA-miRNA-mRNA network

In our study, we used bioinformatics tools to identify several differentially expressed genes (DEGs) from Gene Expression Omnibus (GEO, https://www.ncbi.nlm.nih.gov/gds) and differentially expressed circRNAs (DE-circRNAs) from GC tissue and normal tissue, further constructing a circRNA-miRNA-mRNA network to explore these differentially expressed molecules. Moreover, emerging evidence suggests that ceRNA networks mediate the crosstalk between malignant tumor cells and tumor-infiltrating immune cells (TIICs), significantly influencing the distal survival time of patients. Therefore, we analyzed the potential of the circRNA-miRNA-mRNA network constructed in our study for the prediction of prognosis and immune infiltration. Finally, we preliminarily validated the results via The Cancer Genome Atlas (TCGA, https://tcga-data.nci.nih.gov/) database and qRT-PCR. In general, we constructed ceRNA regulatory networks and identified several prognostic- and immune infiltration-related genes, providing promising prospects for GC monitoring and immunotherapy. The flow chart of the comprehensive bioinformatics analysis is shown in Fig. 1.

## Materials and methods

### Acquisition of expression profiles in the GEO database and Differential Analysis

The mRNA sequencing profiles of GC patients and normal controls were obtained from the GEO database (accession numbers: GSE54129 and GPL570, 111 human gastric cancer tissues and 21 noncancerous gastric tissues, submission date: Jan 16, 2014, last updated: Mar 25, 2019, https://www.ncbi.nlm.nih.gov/geo/query/acc.cgi?acc=GSE54129; GSE79973 and GPL570, 10 pairs of GC tissue and adjacent nontumor mucosa, submission date: Apr 06, 2016 https://www.ncbi.nlm.nih.gov/geo/query/acc.cgi?acc=GSE79973, last updated: Oct 07, 2019) [11]. Differentially expressed circRNAs were screened from GSE163416 (GPL20795, 3 chronic superficial gastritis samples, 3 chronic atrophic gastritis + intestinal metaplasia samples, 3 dysplasia samples and 3 gastric cancer samples, submission date: Dec 17, 2020, last updated: Jul 07, 2021 https://www.ncbi.nlm.nih.gov/geo/query/acc.cgi?acc=GSE163416) [12] and GSE78092 (GPL21485, 3 normal and 3 cancer tissues, submission date: Feb 19, 2016, last updated: Oct 26, 2017 https://www.ncbi.nlm.nih.gov/geo/query/acc.cgi?acc=GSE78092) [13]. All of our data were quantile normalized and the batch effect were eliminated using the ‘normalizeBetweenArrays’ function in ‘limma (version 3.52.2)’ package of R (version 4.2.1) [14].

### Identification of DEGs and DE-circRNAs

We used the GEO2R online analysis tool (https://www.ncbi.nlm.nih.gov/geo/geo2r/) to select DEGs and DE-circRNAs with the threshold of |log2(fold change [FC]) | > 1 and adjusted *p* ≤ 0.05. A Venn diagram was constructed with Venny 2.1 software (http://bioinfogp.cnb.csic.es/tools/venny/) to find the intersecting molecules.

### Construction of the ceRNA Regulatory Network

Above all, we assume that each ceRNA pair is positively correlated with each other and negatively correlated with their shared miRNAs. The target miRNAs for DE-circRNAs were predicted from the Circular RNA Interactome (CircInteractome) (https:/circinteractome.nia.nih.gov/) [15]. The mRNAs binding to miRNAs were predicted via the mirDIP database (http://ophid.utoronto.ca/mirDIP/index_confirm.jsp) [16]. The “bidirectional” mode and all twenty data sources were selected, and three or more of the 20 software programs as well as the top 5% of the confidence class genes (high) were deemed to be possible target genes. The corresponding DE-circRNAs and the bidirectional miRNA-mRNA network were used to establish the circRNA-miRNA-mRNA network by Cytoscape version 3.8.0 software.

### Construction of the protein-protein interaction network

We used the STRING database (https://string-db.org) to generate a protein-protein interaction (PPI) network with interactors of co-DEGs, and a combined score ≥ 0.4 was considered to indicate a significant PPI pair [17–19]. Then, we output the data to Cytoscape software for visualization. The top 15 Hubba DEGs were identified based on the cytoHubba plug-in with the degree algorithm [20]. The MCODE plug-in was applied to filter highly interconnected subclusters [21].

### Gene Ontology and Pathway Enrichment analyzes

Gene Ontology (GO) function analysis and Kyoto Encyclopedia of Genes and Genome (KEGG) pathway enrichment analysis of DEGs and circRNA-miRNA-mRNA networks was performed using the ClusterProfiler version 3.14.3 package and GOplot version 1.02 in R V4.0.3 software [22–24] including biological process (BP), cellular component (CC), and molecular function (MF) terms [25, 26]. Differences with an adjusted *p* ≤ 0.1 were considered meaningful.

### Survival analysis and nomogram of prognosis-related genes

We further investigated the prognostic potential of high-degree hub genes. We downloaded the data from the TCGA database for Kaplan-Meier analysis to draw overall survival time (OS) and disease-free survival time (DSS) curves. The hazard ratio (HR) as well as corresponding 95% confidence intervals were calculated, and *p* < 0.05 was considered statistically significant. We combined some common clinical risk factors for gastric cancer and the expression level of prognosis-related genes to construct a nomogram model to predict the 1-, 3-, and 5-year OSs of GC patients via the nomogram package in R. Meanwhile, the concordance index (C-index) was used to evaluate the discrimination of the nomogram between what the model predicted and that actually observed in the calibration curves.

### Immune infiltration analysis

Tumor IMmune Estimation Resource 2.0 (TIMER2.0, http://timer.cistrome.org/) is an open web server for analysing tumor-infiltrating immune cells in various cancers [27]. We used TIMER2.0 to estimate the association between the prognosis-related genes and immune infiltration. A *p* < 0.05 indicated that the difference was meaningful.

### Specimens and clinical information

All of the tissue and plasma samples included in our study were obtained from the Cancer Center for Gastroenterology, the The First Affiliated Hospital of Ningbo University, China, between 2021 and 2022. Cancer tissues, paired adjacent nontumorous tissues (5 cm away from the edge of the tumor) and plasma were collected from 30 patients who underwent surgical procedures. Thirty healthy tissue and plasma samples were obtained from volunteers who underwent gastroscopy. Tissue samples were immediately immersed in RNA fixer (Bioteke, Beijing, China) after removal and preserved at − 80 °C for further use. Each selected patient provided written informed consent prior to gastroscopy or surgery. All experimental protocols in this study were approved by the Ethics Committee of The First Affiliated Hospital of Ningbo University (No. KY20220101).

### TCGA validation cohort and quantitative real-time PCR (qRT-PCR)

TCGA GC data were used as a validation cohort to verify the expression level of DEGs. RNA from clinical samples was extracted from tissue and plasma using TRIzol reagent or TRIzol LS reagent (Ambion, Carlsbad, CA, USA). Then, total mRNA was used as a template and reverse transcribed to cDNA using a GoScript Reverse Transcription (RT) System (Promega, Madison, WI, USA) following the manufacturer’s instructions [28]. qRT-PCR was performed with GoTaq qPCR Master Mix (Promega) following the manufacturer’s instructions on an Mx3005P Real-Time PCR System (Stratagene, La Jolla, CA, USA). The reaction conditions were as follows: 40 cycles of denaturation at 95 °C for 15 s, annealing at 53 °C for 30 s, and extension at 72 °C for 30 s, followed by a final extension at 72 °C for 7 min. The sequences of the primers are included in Supplementary Table 1. All primers were synthesized by BGI Group (Guangdong, China). The fold change of targeted genes was standardized using the ΔCt method [29]. A higher ΔCt was indicative of a lower expression level. The ROC curves and the corresponding AUC values of the ROC curves were output using GraphPad Prism 9.0 (GraphPad Software, USA).

## Results

### Identification of GC-Related DEGs and DEcircRNAs

**Fig. 2:**
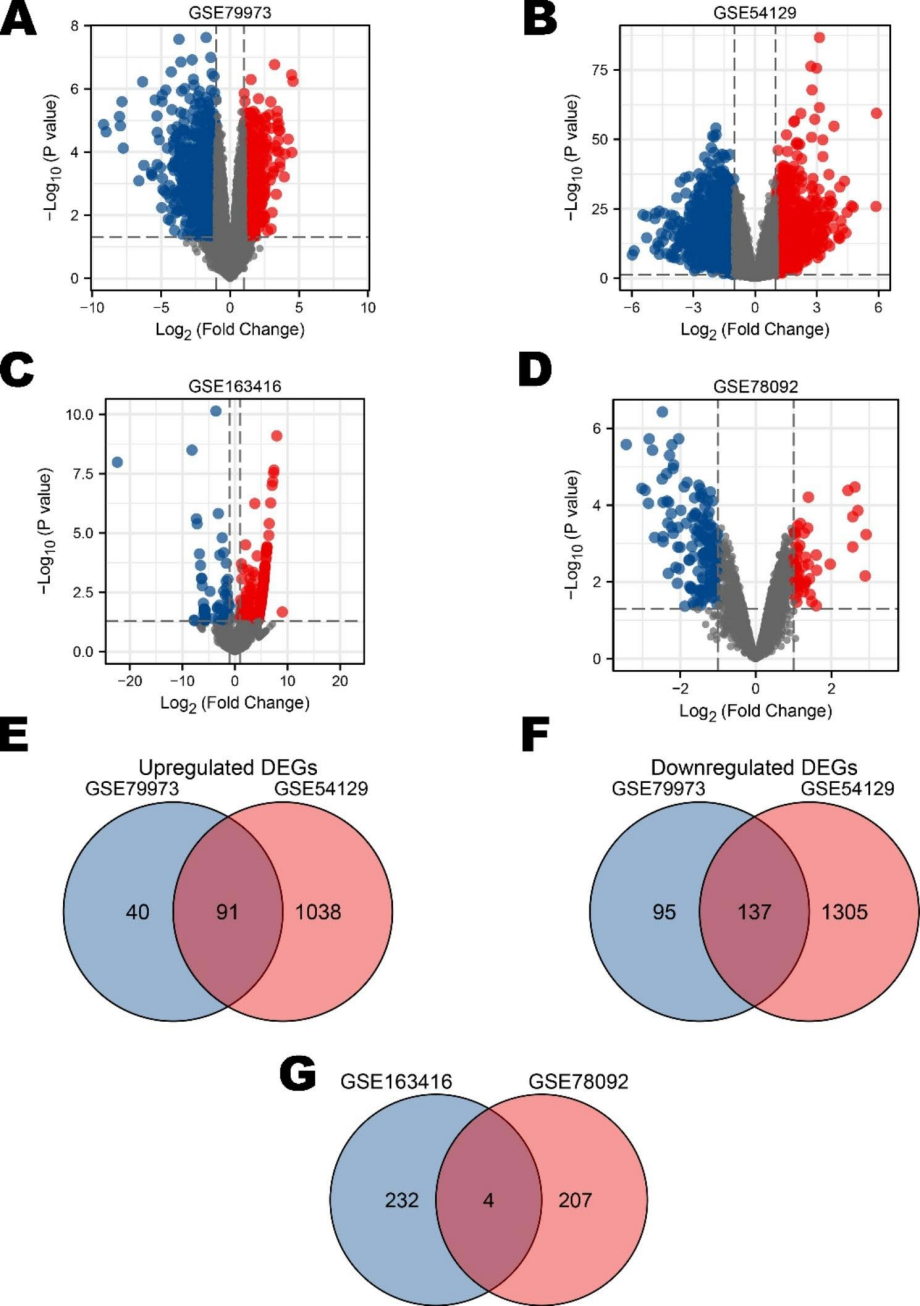
Identification of DEGs and DE-circRNAs by expression profile from the GEO database. (**A**): Volcano plot of 363 DEGs. (**B**): Volcano plot of 2571 DEGs. (**C**): Volcano plot of 236 DE-circRNAs. (**D**): Volcano plot of 211 DE-circRNAs. (**E**): Venn diagram showing 91 upregulated co-DEGs in the mRNA expression profile. (**F**): Venn diagram showing 131 downregulated co-DEGs in the mRNA expression profile. (**G**): Venn diagram showing the presence of 4 codifferentially expressed circRNAs in the circRNA expression profile

Two sets of mRNA expression profiles and two sets of circRNA expression profiles were obtained from the GEO database. We analyzed these data using the GEO2R online tool, and 363 and 2571 DEGs were extracted from GSE79973 and GSE54129, respectively (Fig. 2A and B). A total of 236 and 211 DE-circRNAs were extracted from GSE163416 and GSE78092, respectively (Fig. 2C and D). We divided these DEGs into upregulated and downregulated groups according to logFC and visualized the codifferentially expressed molecules via Venny 2.1 software. There were 91 co-upregulated DEGs and 131 co-downregulated DEGs in GSE79973 and GSE54129 (Fig. 2E F). One DE-circRNA (hsa_circ_0063853) was upregulated, and three DE-circRNAs (hsa_circ_0000673, hsa_circ_0005777, and hsa_circ_0008801) were downregulated (Fig. 2G).

### Establishment of circRNA-miRNA-mRNA networks

**Fig. 3:**
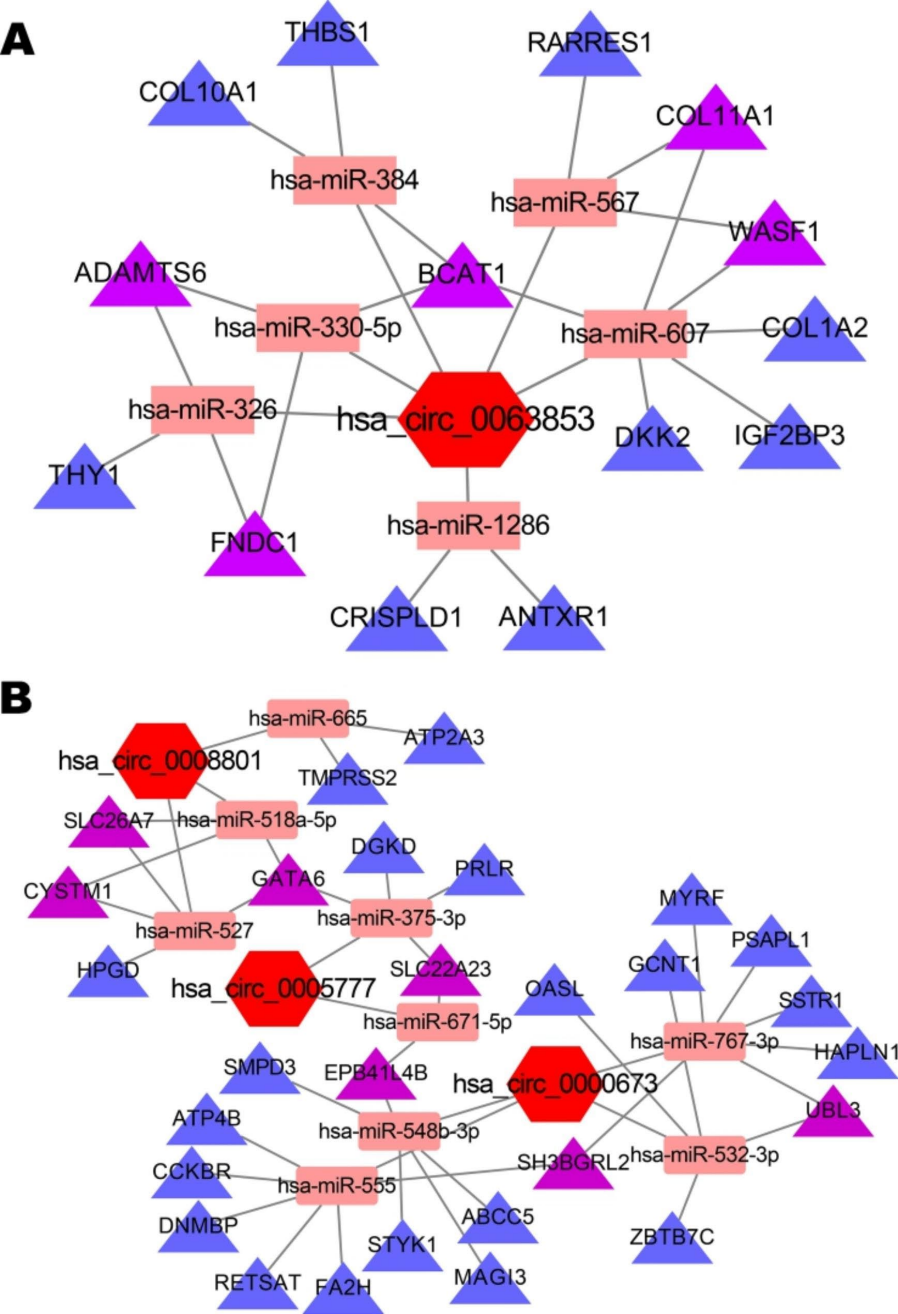
The circRNA-miRNA-mRNA regulatory network in GC. (**A**) The upregulated circRNA-miRNA-mRNA regulatory network contains 21 nodes and 26 edges. (**B**) The downregulated circRNA-miRNA-mRNA regulatory network contains 40 nodes and 44 edges. The red rectangle represents DE-circRNA, the pink hexagons represent miRNAs, the blue triangles represent DEGs and the purple triangles represent cotargeted DEGs.

To comprehend the relationship between DEGs and DE-circRNAs, we needed to establish the circRNA-miRNA-mRNA network. We first searched CircInteractome to ascertain the miRNAs sponged by the identified DE-circRNAs. There were 4 target miRNAs for hsa_circ_0000673, 2 target miRNAs for hsa_circ_0005777, 3 target miRNAs for hsa_circ_0008801 and 6 target miRNAs for hsa_circ_0063853. Then, we used the “bidirectional” mode of the mirDIP database to determine the relationship between miRNAs and mRNAs and visualized the results using Cytoscape software. Our results showed that upregulated hsa_circ_0063853 absorbed 6 miRNAs and 13 upregulated mRNAs, which formed 20 interactive miRNA-mRNA pairs (Fig. 3A). Likewise, 3 circRNAs, 9 miRNAs, and 28 mRNAs formed 36 interactive miRNA-mRNA pairs (Fig. 3B). Interestingly, BCAT1 was simultaneously targeted by 3 miRNAs, and COL11A1, ADAMTS6, WASF1, FNDC1, SLC26A7, CYSTM1, GATA6, SLC22A23, EPB41L4B, SH3BGRL2, and UBL3 were targeted by 2 miRNAs.

### Construction of PPI network and module analysis

**Fig. 4:**
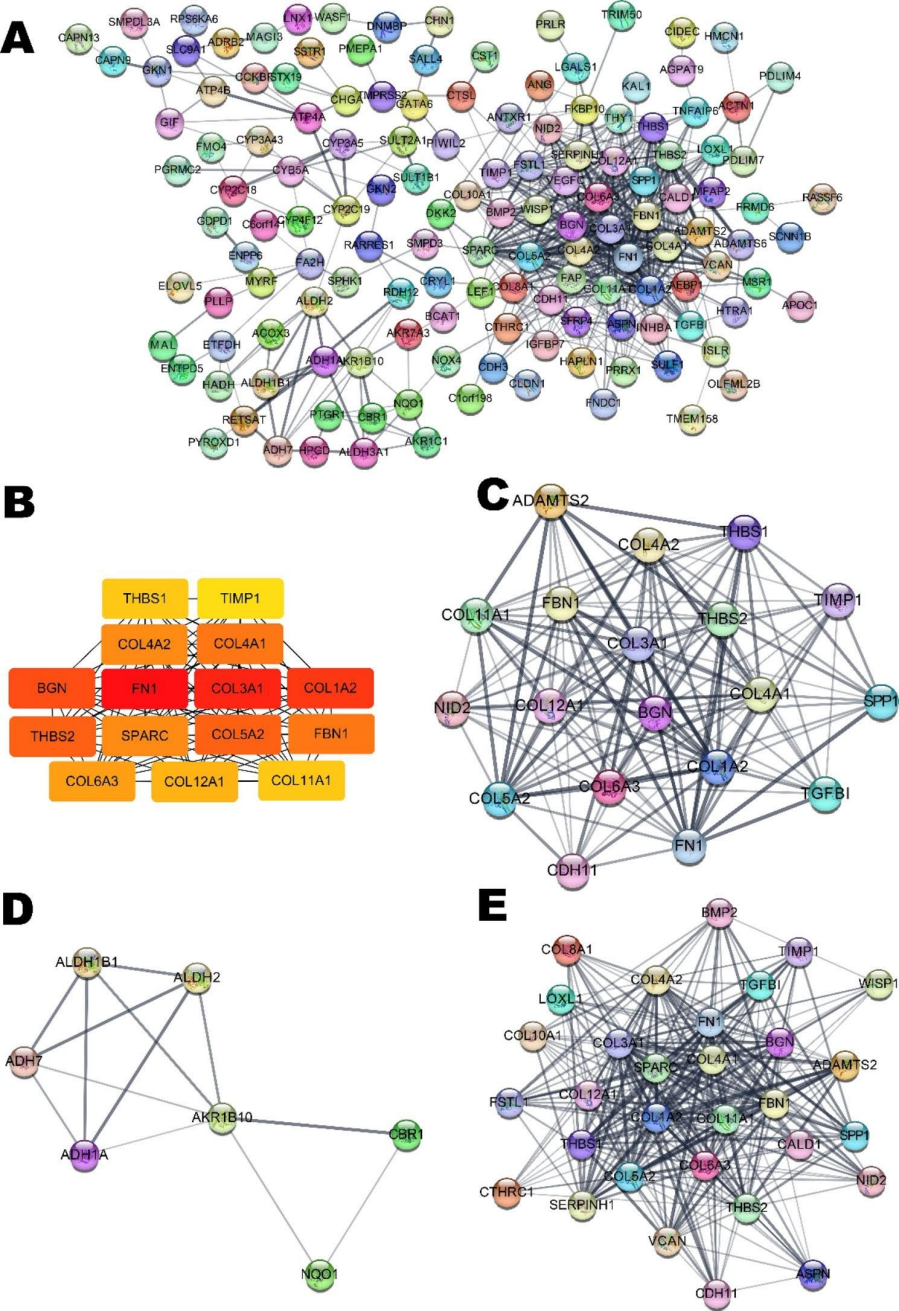
The PPI network of DEGs. (**A**) The PPI network contained 222 nodes and 562 edges from the STRING database. (**B**) The top 15 hub genes with the degree algorithm. A darker colour in the node indicates a higher degree of interaction. (**C**) Module 1 contains 19 nodes and 142 edges. (**D**) Module 2 contains 7 nodes and 13 edges. (E) Module 3 contains 31 nodes and 285 edges

All of the identified DEGs were imported into the STRING database to filter the unpaired proteins and build the DEG interaction diagram. Then, we exported the data to Cytoscape software for polishing and further analysis. The PPI network contained 222 nodes and 562 edges, as shown in Fig. 4A. Next, we used the cytoHubba plug-in in Cytoscape to identify the top 15 genes with node degrees from the PPI network, including FN1, COL3A1, COL1A2, BGN, THBS2, COL5A2, COL4A1, FBN1, COL4A2, SPARC, COL6A3, COL12A1, COL11A1, THBS1, and TIMP1 (Fig. 4B; Table 1). All of them were upregulated, and some were present in the hsa_circ_0063853 ceRNA network, such as THBS1, COL11A1, and COL1A2, implying that these proteins and hsa_circ_0063853 are tightly correlated with the carcinogenesis of GC. Moreover, we used the MCODE plug-in to determine highly interconnected subclusters in the network. Three core modules were obtained, including 19, 7, and 31 nodes and 142, 13, and 285 edges (Fig. 4C-E).

**Table 1:** 15 hub genes from the PPI network

Gene Symbol	Full name	Degree	Expression
FN1	Fibronectin 1	46	Upregulation
COL3A1	Collagen type III alpha 1 chain	40	Upregulation
COL1A2	Collagen type I alpha 2 chain	39	Upregulation
BGN	Biglycan	36	Upregulation
THBS2	Thrombospondin 2	31	Upregulation
COL5A2	Collagen type I alpha 2 chain	31	Upregulation
COL4A1	Collagen type IV alpha 1 chain	28	Upregulation
FBN1	Fibrillin 1	28	Upregulation
COL4A2	Collagen type IV alpha 2 chain	27	Upregulation
SPARC	Secreted protein acidic and cysteine rich	27	Upregulation
COL6A3	Collagen type VI alpha 3 chain	26	Upregulation
COL12A1	Collagen type XI alpha 1 chain	25	Upregulation
COL11A1	Collagen type XII alpha 1 chain	24	Upregulation
THBS1	Thrombospondin 1	24	Upregulation
TIMP1	TIMP metallopeptidase inhibitor 1	22	Upregulation

### Functional enrichment analysis of DEGs and ceRNA networks

**Fig. 5:**
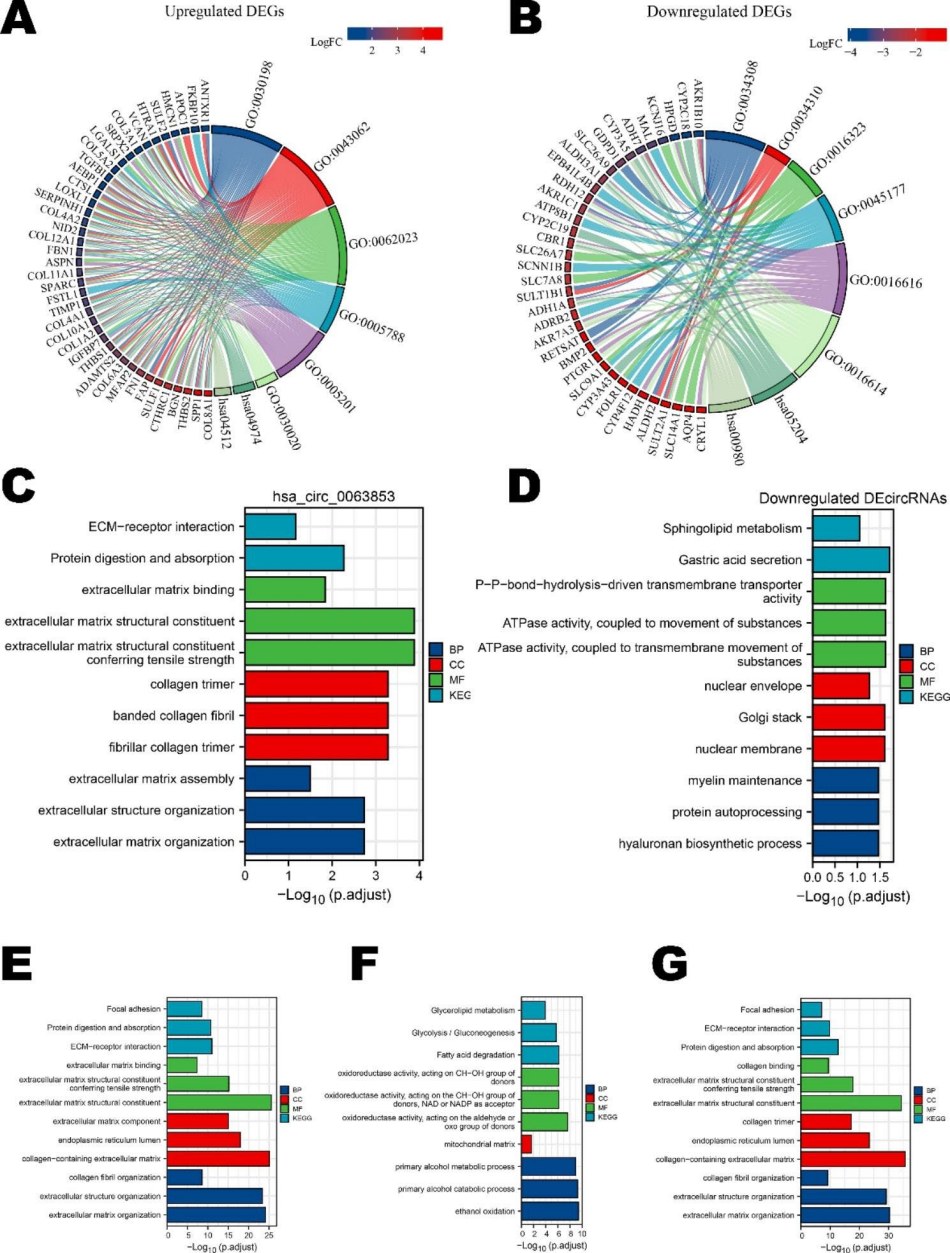
Functional enrichment analysis of DEGs and ceRNA networks. (**A**) Chord diagram of KEGG and GO analyzes of upregulated DEGs. (**B**) Chord diagram of KEGG and GO analyzes of downregulated DEGs. (**C**) KEGG and GO analyzes of the ceRNA network regulated by hsa_circ_0063853. (**D**) KEGG and GO analyzes of the downregulated ceRNA network. (**E-G**) KEGG and GO analyzes of modules 1–3 [22–24]

Next, we performed GO and KEGG functional enrichment analyzes to explore the potential biological function of DEGs and ceRNA networks. We first annotated the functions of the co-DEGs shown in Fig. 5A and B; Tables 2 and 3. The upregulated co-DEGs are tightly associated with extracellular matrix organization and interaction. The downregulated co-DEGs were remarkably correlated with oxidoreductase activities. Then, we investigated the function of the circRNA-miRNA-mRNA networks shown in Fig. 5C and D. Intriguingly, the function of upregulated hsa_circ_0063853 was focused on extracellular matrix and structure, which was in line with the functions of the upregulated co-DEGs, suggesting the close intrinsic connection between these genes. The function of downregulated circRNAs converged on physiological functions, such as protein processing, energy metabolism and gastric acid secretion. Furthermore, we investigated the function of subclusters screened by the MCODE plug-in displayed in Fig. 5E and G. Our results showed that Cluster 1 and Cluster 3 are both associated with the structure of the extracellular matrix. Cluster 2 correlated with the progress of energy metabolism and oxidation. The analysis of MCODE clusters was in accordance with the DE-circRNAs, which revealed that the function of ceRNA networks was closely related to structural changes in the extracellular matrix and cell metabolism in the development of GC.

**Table 2:** The functional enrichment of upregulated DEGs.

Ontology	ID	Description	Gene Ratio	Bg Ratio	P value	p.adjust	q value
BP	GO:0030198	extracellular matrix organization	31/82	368/18,670	5.78e-32	1.08e-28	8.91e-29
BP	GO:0043062	extracellular structure organization	32/82	422/18,670	1.41e-31	1.31e-28	1.09e-28
CC	GO:0062023	collagen-containing extracellular matrix	34/86	406/19,717	4.46e-35	7.58e-33	5.72e-33
CC	GO:0005788	endoplasmic reticulum lumen	21/86	309/19,717	1.45e-19	1.24e-17	9.34e-18
MF	GO:0005201	extracellular matrix structural constituent	26/82	163/17,697	1.59e-33	3.26e-31	2.63e-31
MF	GO:0030020	extracellular matrix structural constituent conferring tensile strength	10/82	41/17,697	2.58e-15	2.65e-13	2.13e-13
KEGG	hsa04974	Protein digestion and absorption	10/43	103/8076	9.88e-11	9.98e-09	8.63e-09
KEGG	hsa04512	ECM-receptor interaction	8/43	88/8076	1.53e-08	7.72e-07	6.68e-07

**Table 3:** The functional enrichment of downregulated DEGs.

Ontology	ID	Description	Gene Ratio	Bg Ratio	P value	p.adjust	q value
BP	GO:0034308	primary alcohol metabolic process	11/116	85/18,670	4.77e-12	8.35e-09	7.84e-09
BP	GO:0034310	primary alcohol catabolic process	5/116	15/18,670	2.43e-08	2.13e-05	1.99e-05
CC	GO:0016323	basolateral plasma membrane	8/124	217/19,717	6.98e-05	0.012	0.012
CC	GO:0045177	apical part of cell	9/124	384/19,717	7.28e-04	0.053	0.052
MF	GO:0016616	oxidoreductase activity, acting on the CH-OH group of donors, with NAD or NADP as the acceptor	13/115	119/17,697	8.08e-13	2.55e-10	2.02e-10
MF	GO:0016614	oxidoreductase activity, acting on the CH-OH group of donors	13/115	128/17,697	2.09e-12	3.30e-10	2.60e-10
KEGG	hsa05204	Chemical carcinogenesis	9/70	82/8076	2.89e-08	3.64e-06	3.25e-06
KEGG	hsa00980	Metabolism of xenobiotics by cytochrome P450	8/70	77/8076	2.77e-07	1.74e-05	1.56e-05

### Prognostic characteristics of RNAs in the ceRNA network

**Fig. 6:**
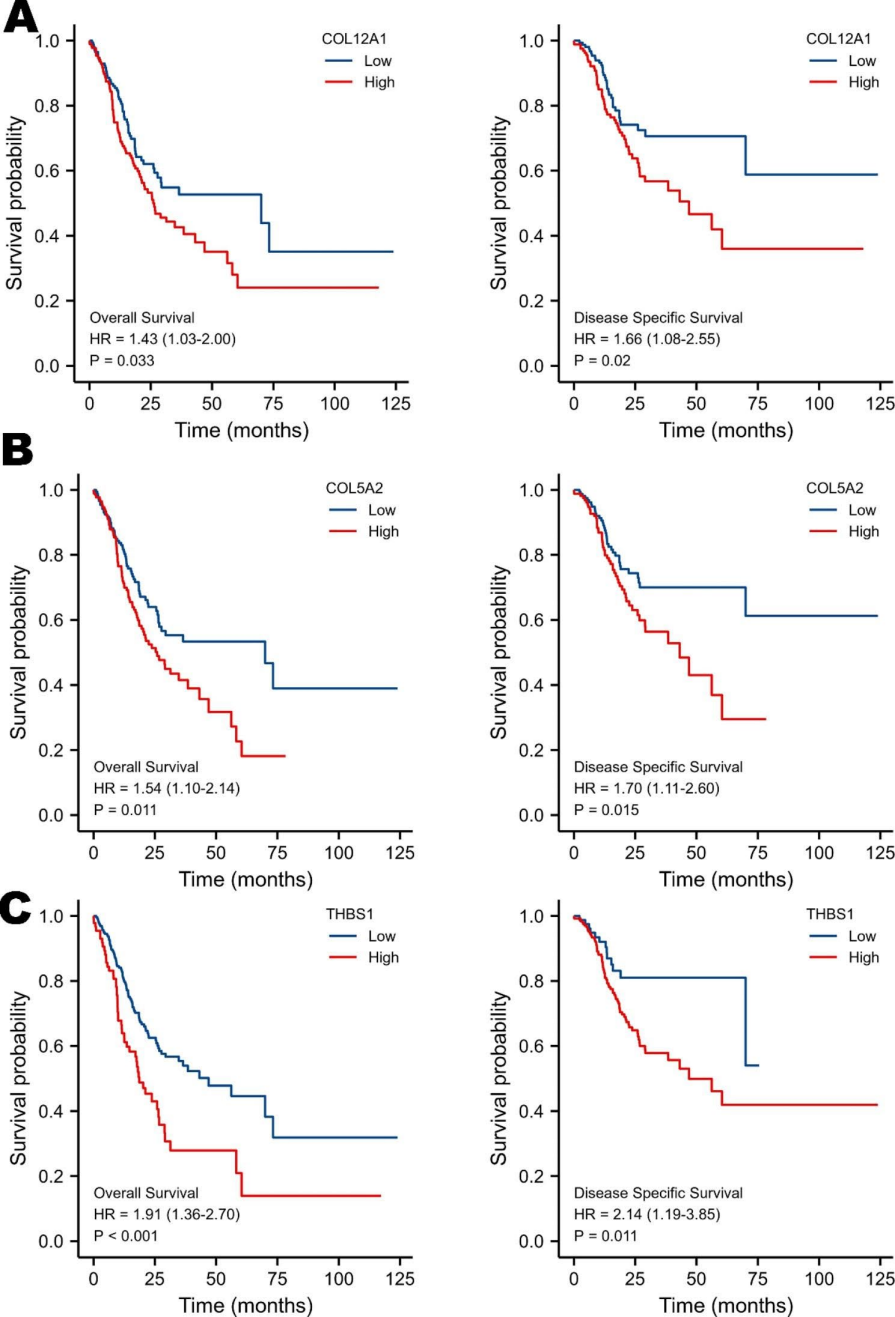
OS and DSS analysis of the top 15 hub genes by the Kaplan-Meier plotter database in GC patient samples from the TCGA database. Survival analysis showing that upregulated expression levels of COL12A1 (**A**), COL5A2 (**B**), and THBS1 (**C**) are correlated with poor survival time (*p* < 0.05)

**Fig. 7:**
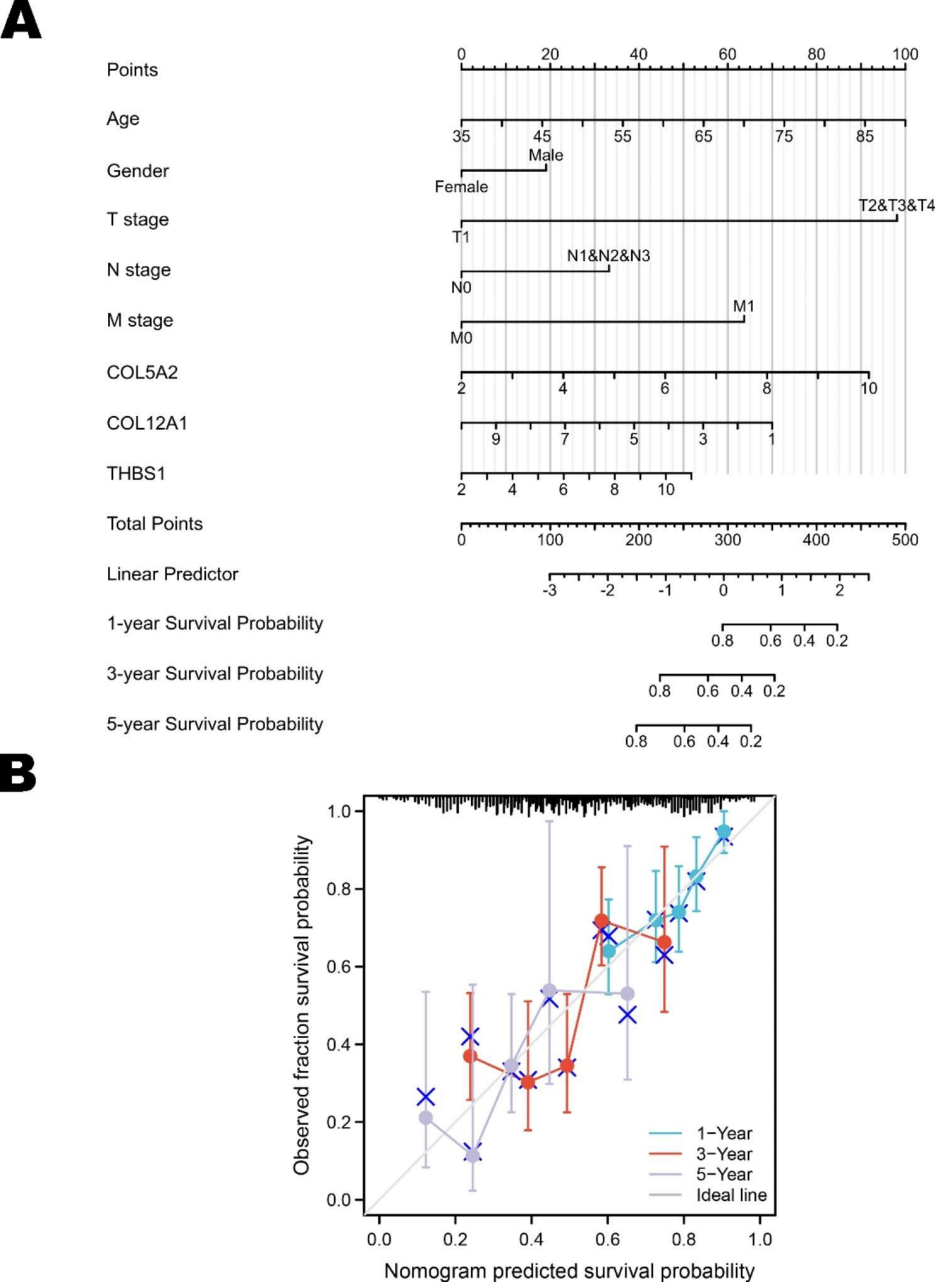
Construction and evaluation of the nomogram model of prognosis-related genes from the TCGA database. (**A**) The nomogram for predicting the 1-, 3-, and 5-year prognosis of GC patients in the TCGA cohort (C-index 0.660). (**B**) The calibration curve demonstrating the discrimination and accuracy of the nomogram for 1, 3, and 5 years

We further investigated the prognostic values of key molecules of the top 15 hub genes in the ceRNA network via the TCGA cohort. The TCGA cohort contains 375 GC tissues and 32 adjacent normal tissues. The results in Fig. 6 show that overexpression of COL12A1, COL5A2, and THBS1 was significantly associated with poor OS and DSS in GC patients. Meanwhile, there was no significant difference between other genes in GC (Supplementary Fig. 1), suggesting that these genes had the potential to be novel biomarkers for GC prognostic prediction. Then, we established the OS nomogram model with common clinicopathologic characteristics of the TCGA GC cohort to predict the 1-, 3-, and 5-year OSs of GC patients, as shown in Fig. 7. A total of 370 samples from the TCGA GC cohort were included in the model, and the C-index was 0.660 (0.634–0.686). The calibration of the model was evaluated with calibration curves, and the calibration curves were closer to the 45° line, which implied that the model was well matched to the standard line.

### Immune infiltration analysis of prognosis-related genes

The level of immune infiltration highly influences the prognosis and conveys different outcomes to conventional therapy [30]. Hence, we explored the relationship of prognosis-related genes and immune cell infiltration using the TIMER 2.0 server, including infiltration of CD4 + T cells, CD8 + T cells, regulatory T cells (Tregs), natural killer cells (NKs), cancer-associated fibroblasts, and myeloid-derived suppressor cells (MDSCs). Our tests showed that our prognosis-related genes were comprehensively associated with the immune infiltration level, as shown in Supplementary Fig. 2.

### Primary validation of the diagnostic performance of key prognostic DEGs

**Fig. 8:**
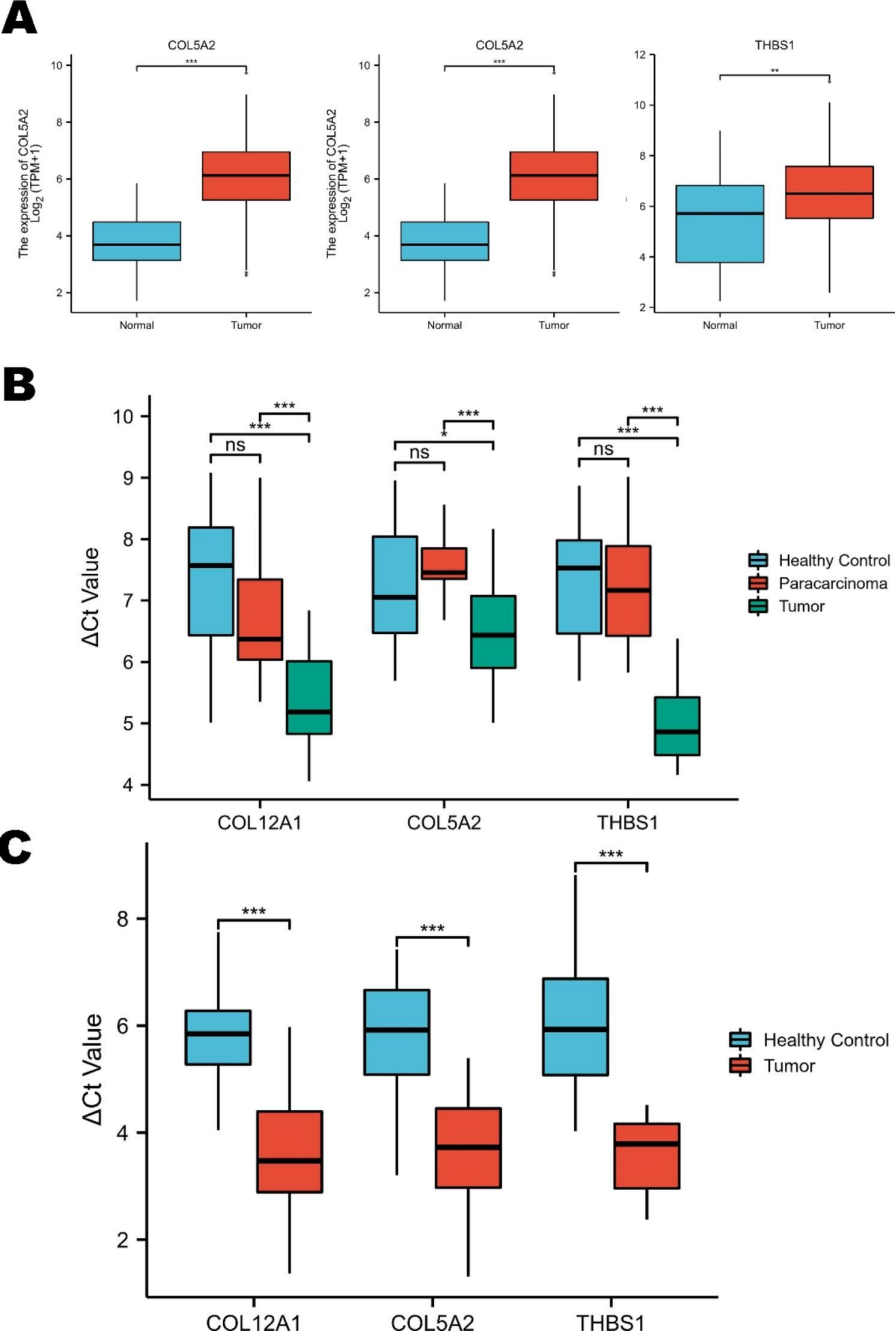
Primary validation of prognostic genes in the TCGA database and clinical samples. (**A**) The expression levels of COL12A1, COL5A2, and THBS1 were significantly upregulated in GC. (**B**) The expression levels of COL12A1, COL5A2, and THBS1 were significantly upregulated in clinical GC tissues. (**C**) The expression levels of COL12A1, COL5A2, and THBS1 were significantly upregulated in clinical GC plasma (* *p* < 0.05, ** *p* < 0.01, *** *p* < 0.001)

As previously mentioned, we screened the DEGs from the GEO database and their prognostic potential from the TCGA database. Therefore, we continued to estimate the differentially expressed level from the TCGA cohort. We found that COL12A1, COL5A2, and THBS1 genes were prominently upregulated in GC tissues (Fig. 8A), which was identical to the findings in the GEO database. Subsequently, the qRT-PCR results also showed that these genes were overexpressed in GC tissues and plasma (Fig. 8B-C). The ROC curve of plasma is shown in Supplementary Fig. 3. The AUC values of COL12A1, COL5A2, and THBS1 were 0.918, 0.934 and 0.968, respectively.

## Discussion

To date, GC is one of the most common and lethal cancers worldwide and is highly heterogeneous [31]. As limited by experimental conditions and the complex pathologic processes of GC, the mechanisms of GC tumorigenesis and development are still ambiguous. Thanks to the conjoint advances in high-throughput sequencing technology and bioinformatic analysis, a large number of significantly differentially expressed RNAs have been identified between tumor and normal tissues, providing new insights into the molecular mechanisms of malignant tumors. CircRNAs are a group of novel endogenous RNAs that play antitumor or tumor-promoting roles in various malignant tumors in several ways, such as acting as miRNA sponges, RNA binding proteins, and regulators of protein translation [32]. Moreover, the stable internal structure of circRNAs makes them excellent candidate tumor biomarkers. For example, hsa_circ_0086720 was proven to be actively secreted by gastric cells and to be stable in circulating plasma and in gastric tumorigenesis; thus, it is a potential biomarker with satisfactory sensitivity and specificity in GC screening and prognostic prediction [33]. Circ_0001190 serves as a sponge for miR-586 and upregulates the expression level of SOSTDC1, effectively mediating the progression of GC [34]. Since the regulatory network of circRNA-miRNA-mRNA is a well-recognized major mechanism in tumor regulation, we used bioinformatic tools to explore the roles of circRNAs in GC in this study.

We first downloaded and analyzed the mRNA and circRNA expression profiles from the GEO database. However, the GEO datasets did not include detailed information, and some confounding factors may be unavoidable. Hence, we give preference to the consensus dataset of references and larger sample sizes. Meanwhile, GSE163416 contains several stages of the progression of gastric cancer, so we also chose this dataset for our study. We screened 91 coupregulated DEGs, 131 codownregulated DEGs, 1 coupregulated circRNA (hsa_circ_0063853), and 3 codownregulated circRNAs (hsa_circ_0000673, hsa_circ_0005777, and hsa_circ_0008801). There are no prior studies reporting the relationship between these circRNAs and GC. We used CircInteractome and mirDIP to define the miRNA-mRNA pairs and established ceRNA networks. We found that some genes were mutually targeted to several miRNAs, suggesting that multiple signal pathways exist to regulate these targets; thus, these genes could be highly suitable for target drug design [35].

Meanwhile, we constructed a PPI network to evaluate the DEGs at the protein level. As a whole, there were several “hub” regions with a high degree of connections with other genes called hub genes, which were not only structural hubs but also functional hubs and participated in a large number of functional interactions in a network [36]. Therefore, it is vital to locate the positions of these hub genes and relevant modules. We filtered the top 15 hub genes by the cytoHubba plug-in and core subclusters by the MCODE plug-in. Then, we annotated these networks and clusters via GO and KEGG functional enrichment analyzes. Possibly the most striking finding was that the hub genes were mainly from the upregulated hsa_circ_0063853 network, and the biological functions of the hub genes, MCODE clusters 1 and 3, and upregulated hsa_circ_0063853 were consistently focused on the extracellular matrix and structure. This highly centralized consistency implied that the upregulated hsa_circ_0063853 network played crucial roles in the progression of GC development. The function of downregulated circRNAs converged on physiological functions, energy metabolism and gastric acid secretion. All these annotations have distinguished theoretical support for GC.

Next, we identified and confirmed prognosis-related genes via the TCGA cohort. Elevated THBS1 is related to liver metastasis and poor prognosis in colorectal cancer patients [37]. Huang et al. revealed that upregulated THBS1 promoted GC cell invasion and migration by fibroblast growth factor 7 and fibroblast growth factor receptor 2, indicating the important role of THBS1 in GC [38]. Lower COL12A1 could inhibit the proliferation and migration of colorectal cancer cells, which may be a new biomarker for prognosis [39]. Similarly, Xiang et al. noted that COL12A1 promoted GC cell migration through positive feedback sustained by the MAPK pathway [40]. A recent study demonstrated that COL5A2 was a key part of tumor progression in colorectal cancer and was associated with poor prognosis in these patients [41]. Tan et al. found that elevated COL5A2 was associated with higher migration and metastasis ability [42]. These studies strongly suggested the prognostic potential of these genes in GC. Hence, we built a nomogram model for prognosis prediction and evaluated it with calibration curves, which was significantly accurate and worthy of prospective, multicentre validation in the future.

Immune cell infiltration is the basis for effective immunotherapy and plays a crucial role in the prognosis of various malignancies [43]. For instance, upregulation of THBS1 has been shown to be correlated with immunity and chemotherapy resistance in GC [44]. Cancer-associated fibroblasts facilitate tumor desmoplasia, becoming a physical obstacle for drug delivery and further reducing the efficacy of chemotherapy and immunotherapy [45]. Recently, research has shown that greater infiltration of Tregs is a huge barrier for immunotherapy and that the depletion of Tregs improves anticancer treatments [46, 47]. Meanwhile, elevated infiltration of Tregs is dependent on the presence of CD8 + T cells, both of which lead to immune-mediated destruction and a tumor escape microenvironment [48]. Thus, we investigated the immune cell infiltration level of these prognosis-related genes. Our results found that most of these genes were accompanied by different levels of immune cell infiltration, which could participate in the formation of a disordered immune microenvironment and influence the distal survival time.

Finally, we preliminarily verified the dysregulation of co-DEGs using the TCGA cohort and qRT-PCR. Our results showed that COL12A1, COL5A2, and THBS1 were overexpressed in GC and that plasma COL12A1, COL5A2, and THBS1 were potential screening biomarkers. These findings further verified our results regarding the dysregulation of co-DEGs, as we mentioned before. However, there are still some deficiencies in this study. The samples to extract mRNA, miRNA and circRNAs were different. Insufficient clinical samples were available in our study, and the function of the network needs further validation.

## Conclusion

In conclusion, we constructed two circRNA-miRNA-mRNA regulatory networks.

We identified 3 GC prognostic and screening biomarkers, COL12A1, COL5A2, and THBS1. The ceRNA network and these genes could play important roles in GC development, prognosis and diagnosis.

## Electronic supplementary material

Below is the link to the electronic supplementary material.

Supplementary Material 1

Supplementary Material 2

Supplementary Material 3

Supplementary Material 4

## Data Availability

The datasets that support the findings of the current study are available in the TCGA, [https://tcga-data.nci.nih.gov/], GTEx, [https://www.gtexportal.org/home/index.html] and GEO [https://www.ncbi.nlm.nih.gov/gds] databases. The datasets analyzed during the current study are available in the “Baiduyun” repository [https://pan.baidu.com/s/1tO3nk19CmEDx_DR1NaDiYA?pwd=9adu]. The data that support the findings of this study are available from the corresponding author upon reasonable request.
