# Supplementary material for: Identification of novel prognostic circRNA biomarkers in circRNA-miRNA-mRNA regulatory network in gastric cancer and immune infiltration analysis
Source: BMC Genomics. 2023 Jun 13;24:323. doi: 10.1186/s12864-023-09421-2 (PMC10262520; doi:10.1186/s12864-023-09421-2)
Supplement: Supplementary file 1 — Supplementary Material 1 [file 12864_2023_9421_MOESM1_ESM.docx]

Supplementary Table 1. Forward and reverse primers listed for qRT-PCR.

| Name | Sequence (5′-3′) |
| --- | --- |
| COL12A1 | F: CCACAGGTTCAAGAGGTCCC |
|  | R: TGTGTTAGCCGGAACCTGGA |
| COL5A2 | F: AACATCAGTTGGGTGGAG |
|  | R: CTTGAAATCGGTGTAGGC |
| THBS1 | F: GCTCCAGTCCTACCAGTGTC |
|  | R: TCAGTCACTTGCGGATGCT |
| GAPDH | F: ACCCACTCCTCCACCTTTGAC |
|  | R: TGTTGCTGTAGCCAAATTCGTT |
