# Supplementary material for: Identification of novel prognostic circRNA biomarkers in circRNA-miRNA-mRNA regulatory network in gastric cancer and immune infiltration analysis
Source: BMC Genomics. 2023 Jun 13;24:323. doi: 10.1186/s12864-023-09421-2 (PMC10262520; doi:10.1186/s12864-023-09421-2)

Supplementary Figure 2 Relationship pf the immune cell infiltration level and prognostic related genes in GC (A) The expression of COL5A2 was positively associated to the infiltration level of CD4+T cell, CD8+T cell, cancer associated fibroblast cell, NK cell and negatively associated to Tregs. (B) The expression of COL12A1 was positively associated to the infiltration level of CD4+T cell, CD8+T cell, cancer associated fibroblast cell, NK cell and negatively associated to Tregs. (C) The expression of THBS1 was positively associated to the infiltration level of CD4+T cell, CD8+T cell, cancer associated fibroblast cell, MDSC, NK cell and negatively associated to Tregs (P < 0.05).
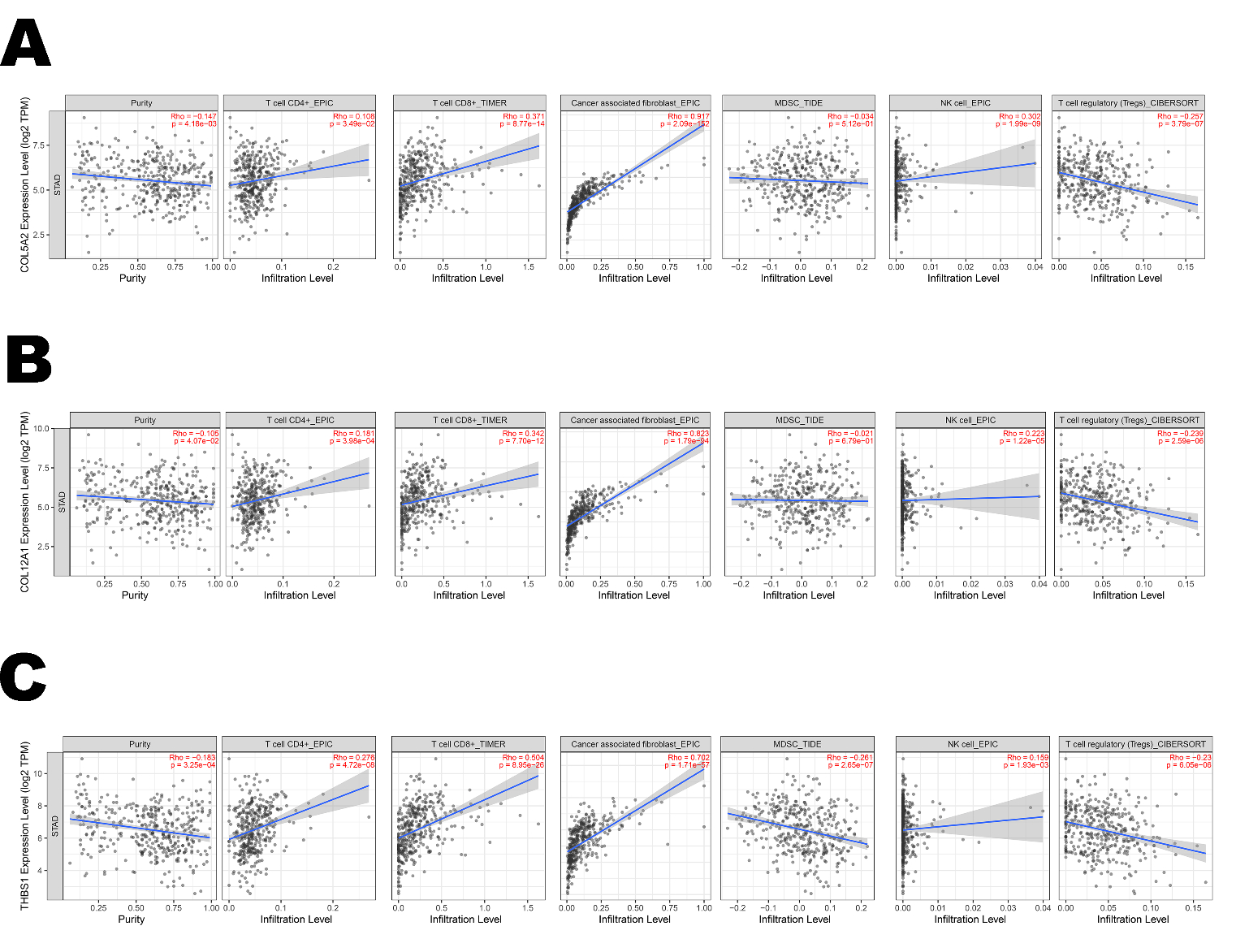

Supplement: Supplementary file 2 — Supplementary Material 2 [file 12864_2023_9421_MOESM2_ESM.docx]
