# Supplementary figures and images for: Identification of novel prognostic circRNA biomarkers in circRNA-miRNA-mRNA regulatory network in gastric cancer and immune infiltration analysis
Source: BMC Genomics. 2023 Jun 13;24:323. doi: 10.1186/s12864-023-09421-2 (PMC10262520; doi:10.1186/s12864-023-09421-2)

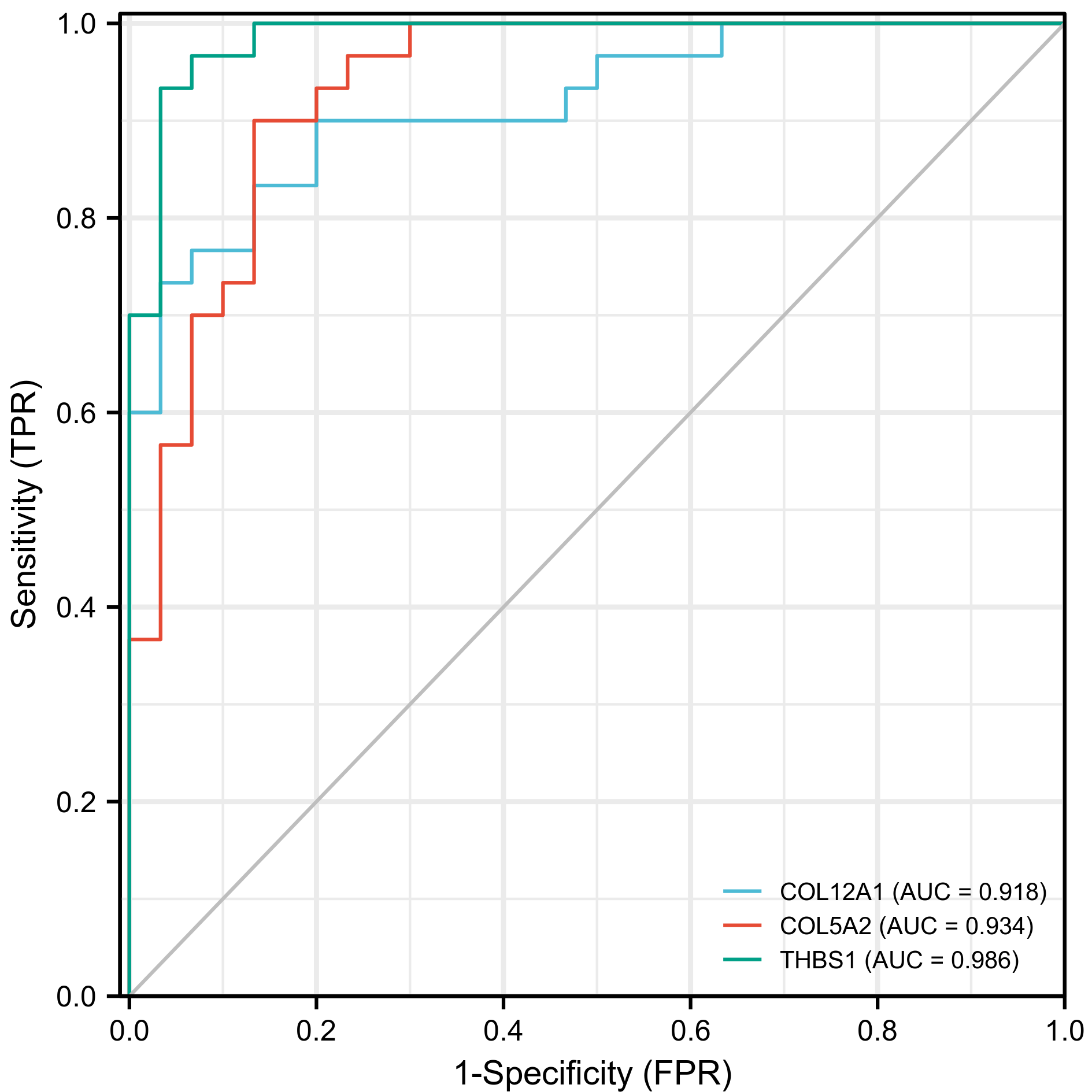


Supplementary Figure 3 The ROC curve of COL12A1, COL5A2, THBS1.

Supplement: Supplementary file 3 — Supplementary Material 3 [file 12864_2023_9421_MOESM3_ESM.docx]

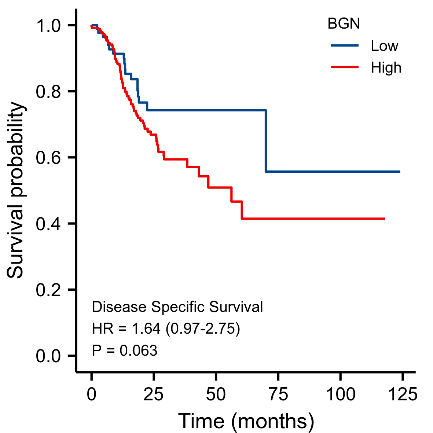

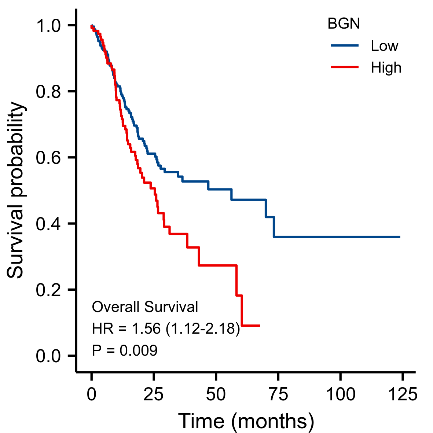


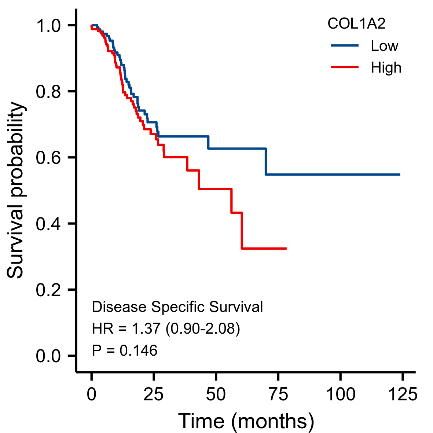

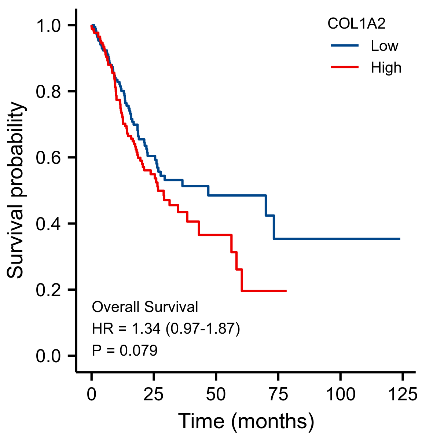


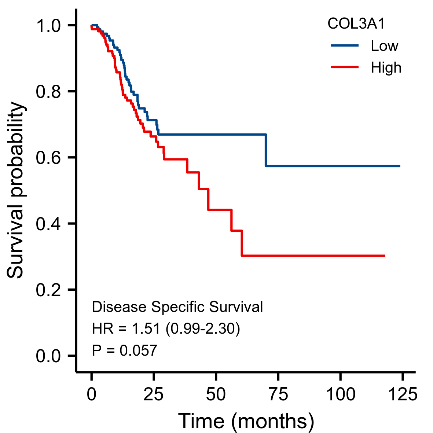

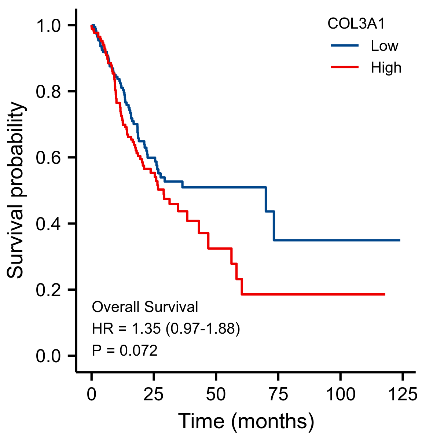


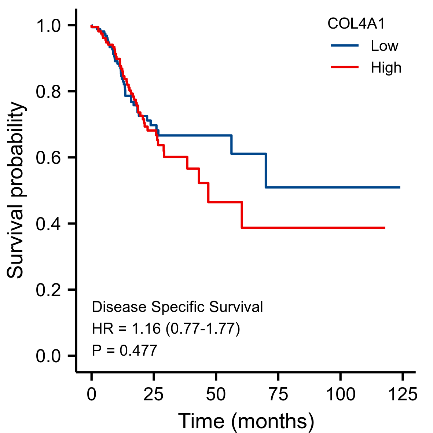

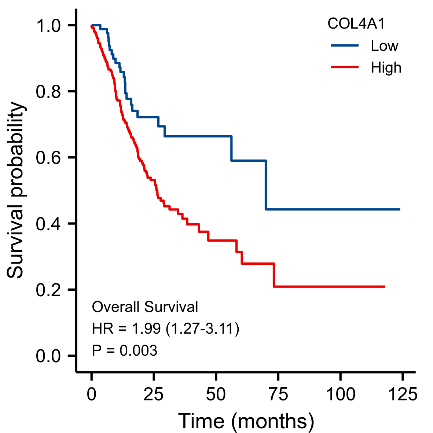


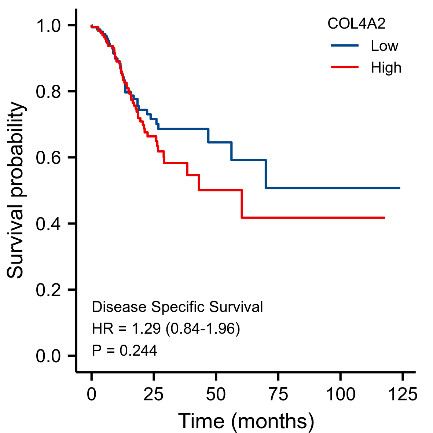

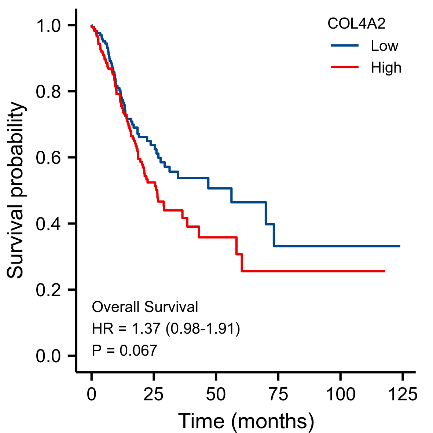


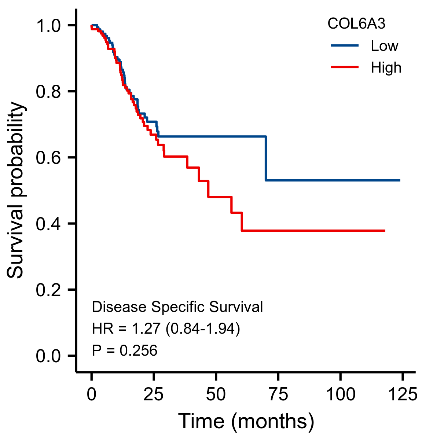

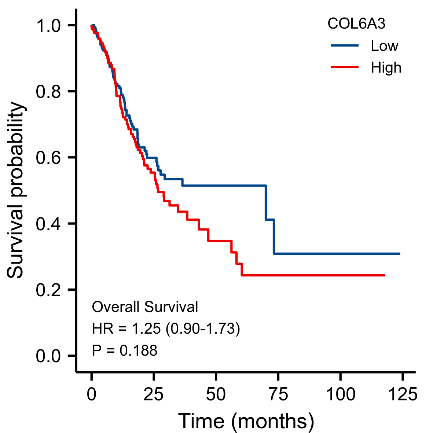


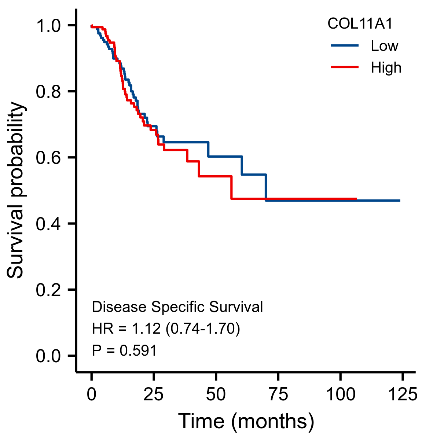

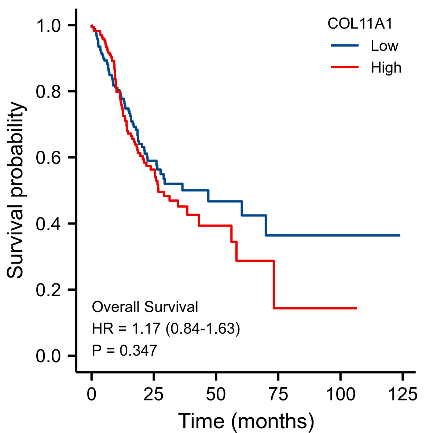


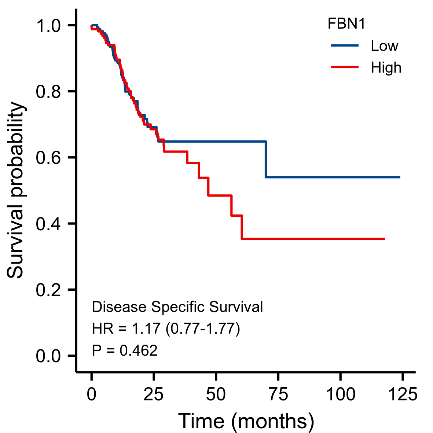

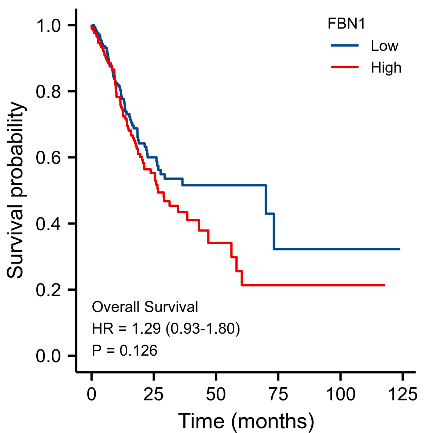


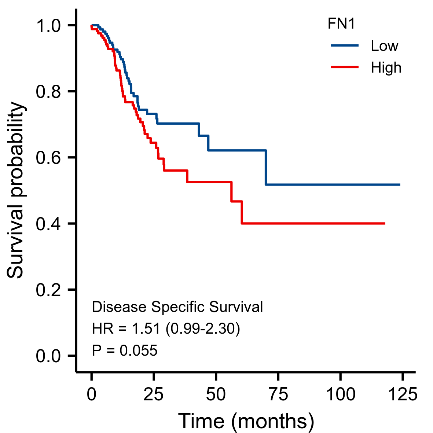

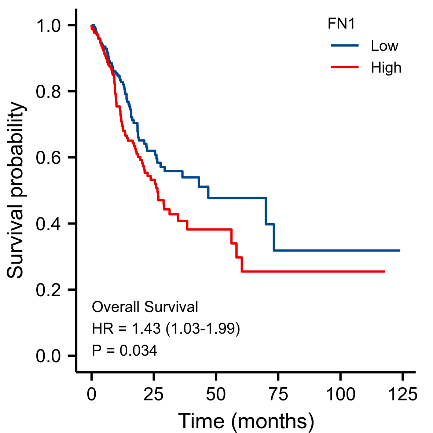


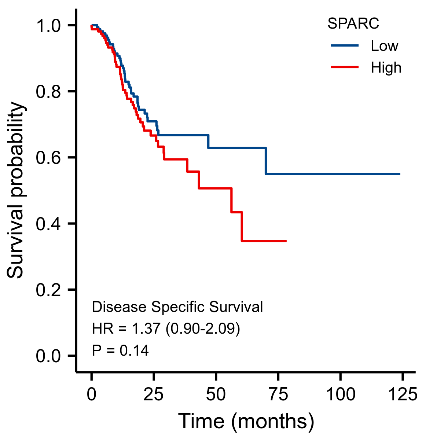

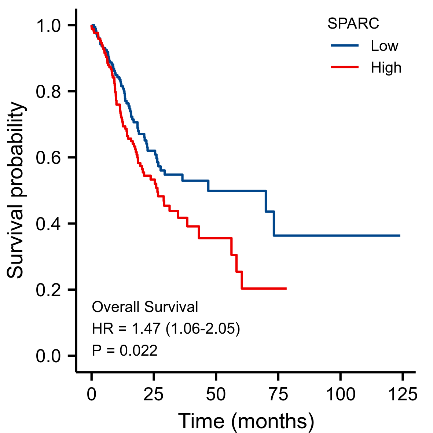


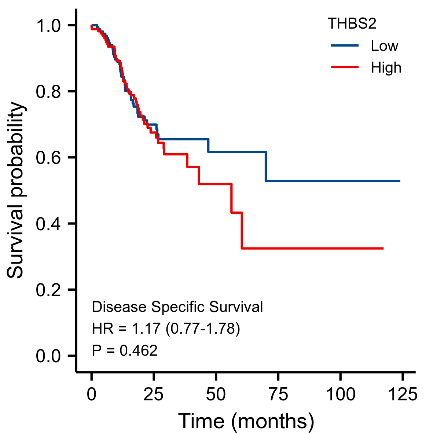

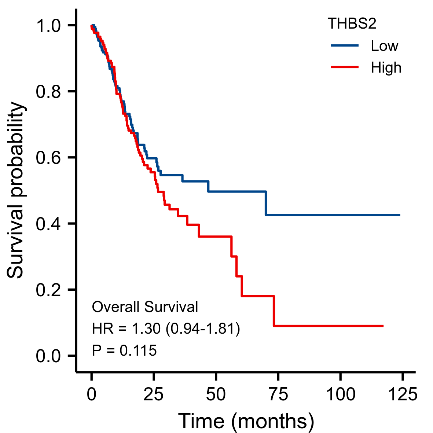


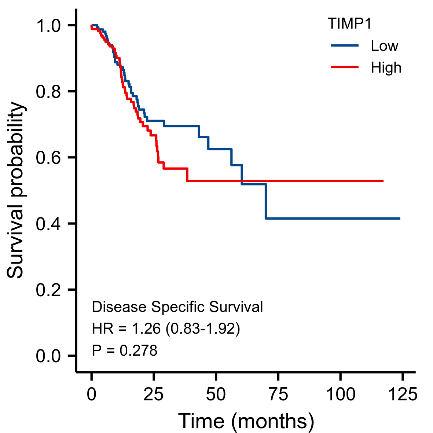

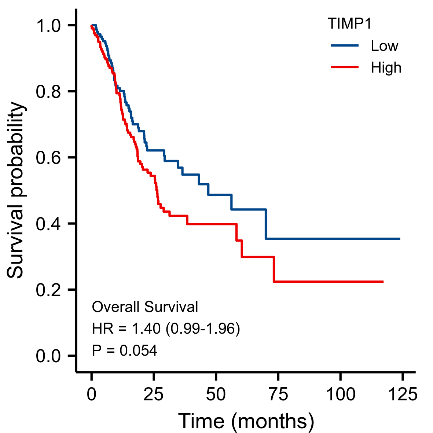


Supplementary Figure 1 The Kaplan-Meier analysis of 15 hub genes

Supplement: Supplementary file 4 — Supplementary Material 4 [file 12864_2023_9421_MOESM4_ESM.docx]
